# Arterial Occlusion and Acute Deep Vein Thrombosis-Induced Acute Limb Ischemia in a COVID-19 Patient

**DOI:** 10.7759/cureus.26689

**Published:** 2022-07-09

**Authors:** Alexander Nguyen, Abe Al Hage, Han Yu, Satheesh Gunaga

**Affiliations:** 1 Department of Emergency Medicine, Henry Ford Health, Wyandotte, USA

**Keywords:** thromboembolism, arterial occlusion, deep vein thrombosis (dvt), acute limb ischemia, covid-19

## Abstract

Coronavirus disease 2019 (COVID-19) is a viral illness known to elicit a hypercoagulable state leading to a myriad of vascular pathologies. Over the course of the COVID-19 pandemic, widespread insults to the venous system have been well documented, with an increasing number of arterial events being reported. Despite the rising incidence of both pathological manifestations, these events are rare, but when present, serve as significant life threats to the patient in question. We report and discuss a case of a 69-year-old female with no thromboembolic risk factors or systemic signs of illness who presented with signs and symptoms consistent with acute limb ischemia (ALI). The patient was ultimately found to have occlusion of multiple arterial and venous vessels. She tested positive for COVID-19 despite being otherwise asymptomatic from a viral syndrome standpoint. To our knowledge, there are no reports in the medical literature of ALI - in the setting of arterial occlusion and concomitant deep vein thrombosis (DVT) - as the sole clinical manifestation in an asymptomatic patient without thrombotic risk factors who was only incidentally found to be COVID-19-positive. This case underscores the atypical manifestations and deleterious effects associated with COVID-19 and the need to have a high index of suspicion for a multitude of pathologies when facing this viral illness.

## Introduction

Coronavirus disease 2019 (COVID-19) is a viral respiratory tract infection responsible for numerous thrombotic events of the major vessels in the human body, most notably deep vein thrombosis (DVT) and pulmonary embolism [1]. Such thromboembolic events are thought to arise from an immune and pro-inflammatory response resulting in the production of procoagulation factors responsible for vascular injury. Despite the overwhelming preponderance of venous etiologies, rare reports now implicate COVID-19 in adverse arterial events such as arterial thrombosis which can lead to acute limb ischemia (ALI) [1]. ALI is an emergent vascular event resulting in a decrease in limb blood flow and subsequent tissue hypoperfusion [2].

This article was previously presented as a poster at the Michigan State University Statewide Campus System Emergency Medicine Resident Case Report Poster Day on February 16, 2022, and at the Henry Ford Health System Medical Education Research Forum on April 1, 2021.

## Case presentation

A 69-year-old female with a past medical history (PMH) significant for type 2 diabetes mellitus well controlled with metformin, presented to the emergency department (ED) with acute onset left lower extremity (LLE) pain that began approximately eight hours prior to arrival. The patient described the pain as burning and throbbing and rated 10/10. She denied all other symptoms, including, but not limited to, upper respiratory symptoms, nausea, vomiting, chest pain, dyspnea, cough, and abdominal pain.

**Figure 1:**
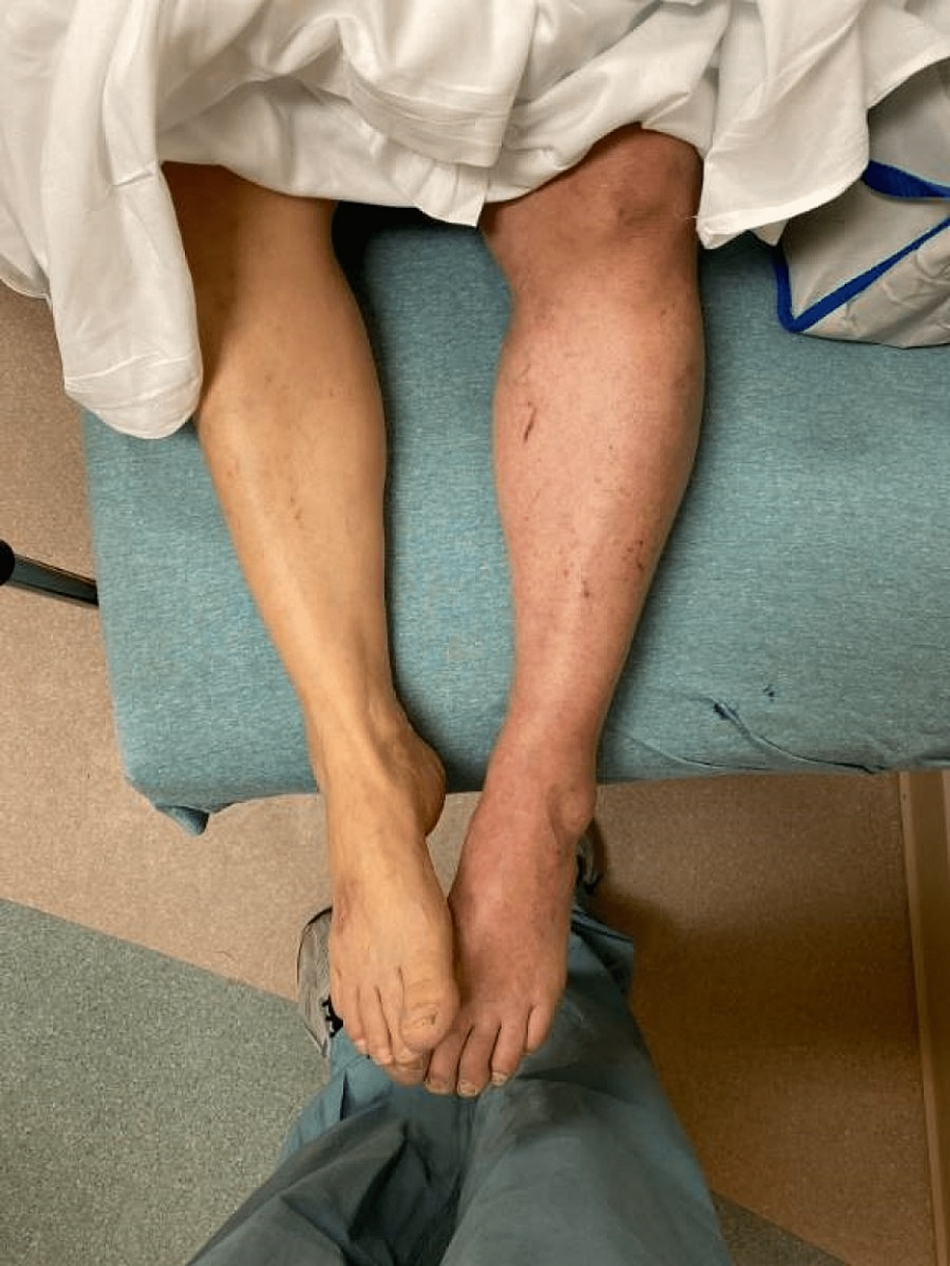
Grossly abnormal left lower extremity highly concerning for acute limb ischemia

She lived an active, non-sedentary lifestyle, was a lifetime non-smoker, and possessed a normal body mass index. She denied a history of atrial fibrillation, blood clots, or known coagulopathies in herself or her family. She had received no COVID-19 vaccinations. Vital signs demonstrated the following: blood pressure was 183/91, heart rate was 90 beats per minute, respiratory rate was 16 breaths per minute, oxygen saturation was 98% on room air, and was afebrile. Electrocardiogram showed normal sinus rhythm with no ischemic changes or abnormalities. Her physical examination was significant for LLE swelling, discoloration with notable erythema and mottling, and excruciating tenderness with palpation (Figure 1). No palpable pulses of the LLE were appreciated at any site, including with the use of bedside Doppler ultrasound; findings highly concerning for ALI. There were no obvious deformities, signs of injury, or wounds appreciated and pulses of the right lower extremity were grossly intact. 

The patient’s initial laboratory workup was also unremarkable - complete blood count, basic metabolic panel, and coagulation panel revealed no derangements of note. The patient was tested for COVID-19 as required for hospital admission and was incidentally found to be COVID-19 polymerase chain reaction (PCR) RNA positive. She again denied symptoms outside of her chief complaint or known exposures. A heparin infusion was initiated in the ED and she was emergently transferred to a tertiary care hospital for further evaluation by a vascular surgery team and escalation of care.

**Figure 2:**
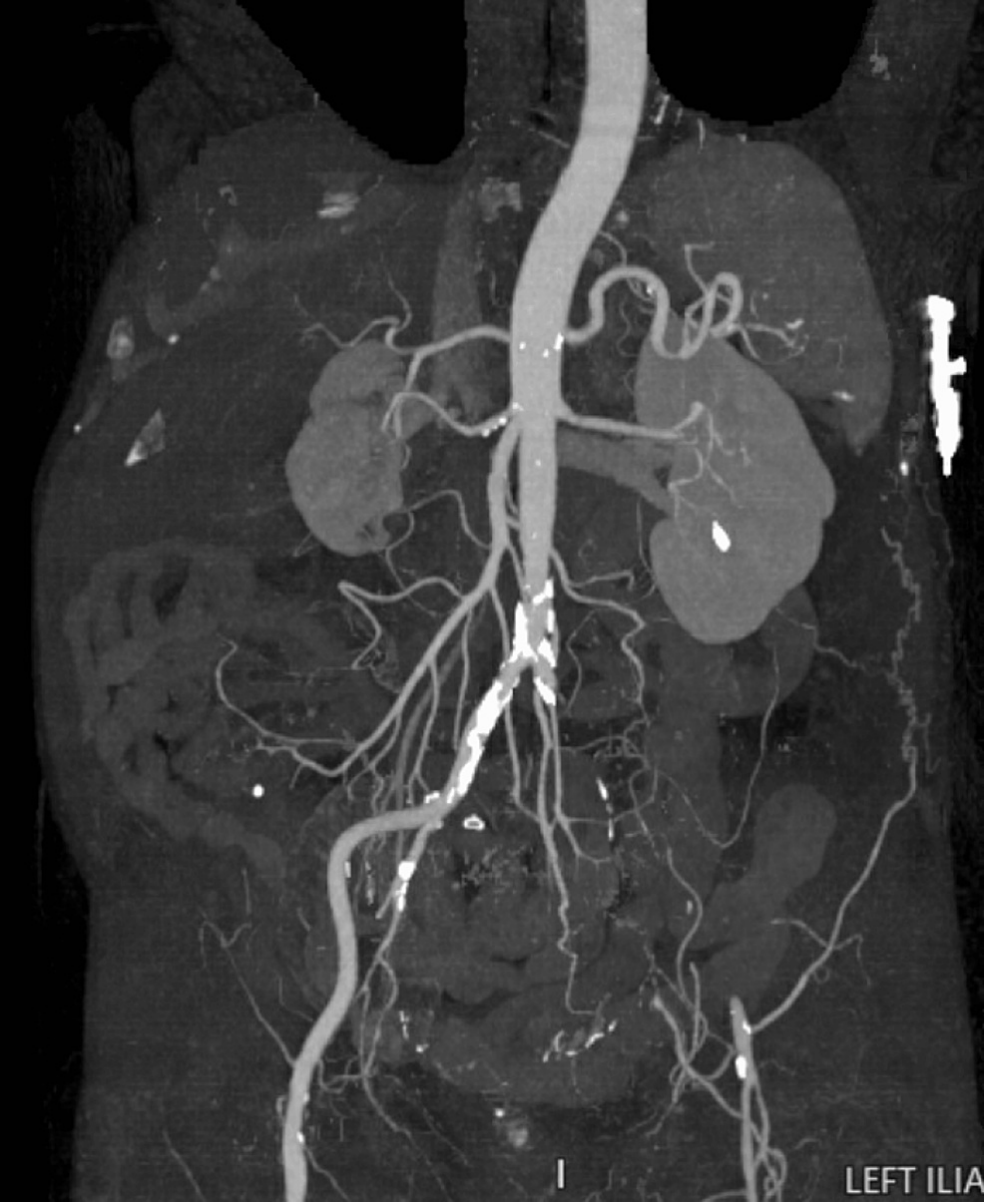
CT angiography imaging demonstrating significant left-sided vascular occlusion

**Figure 3:**
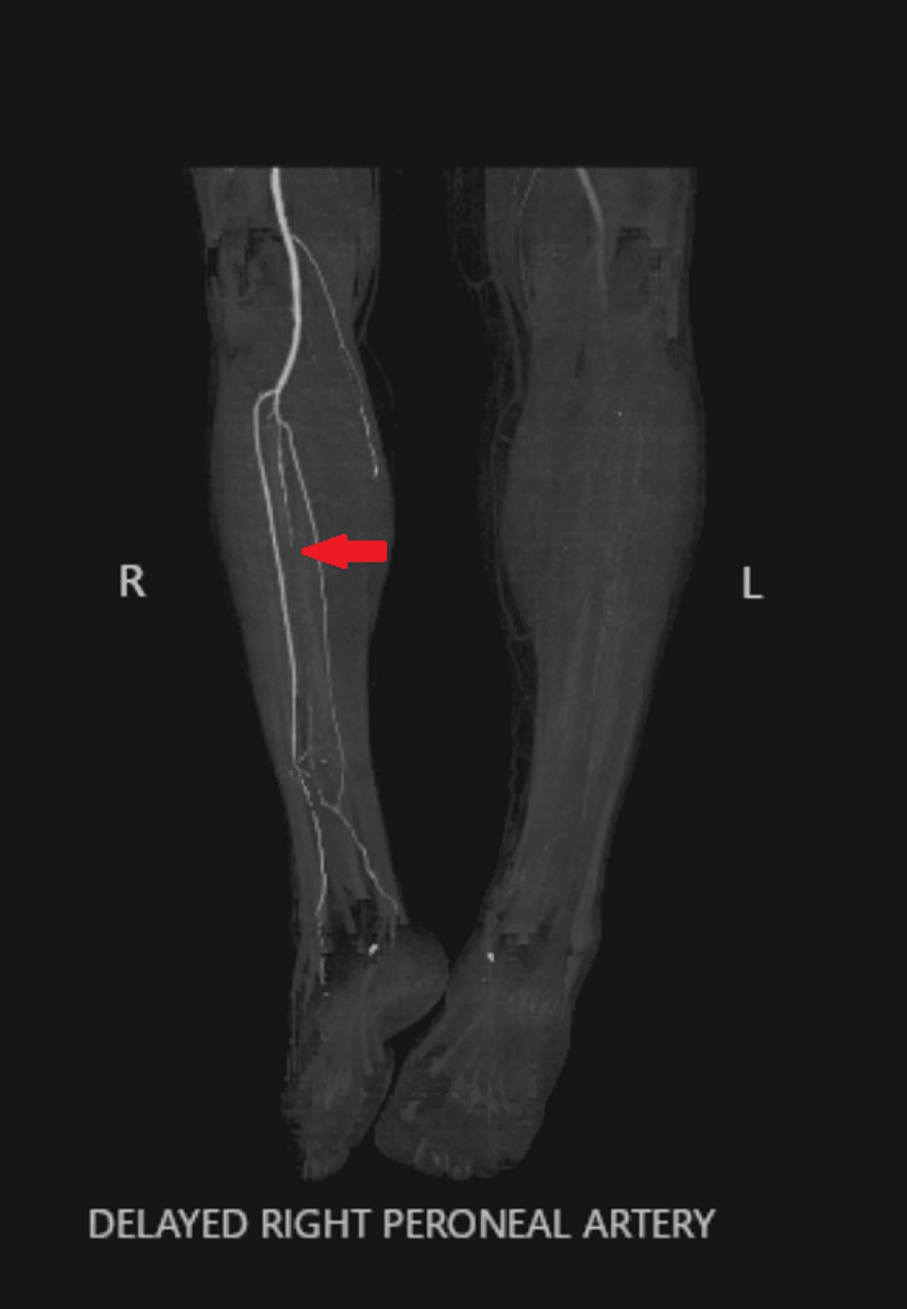
CT angiography imaging demonstrating occlusion of the right peroneal artery in the mid leg at the level of the arrow

**Figure 4:**
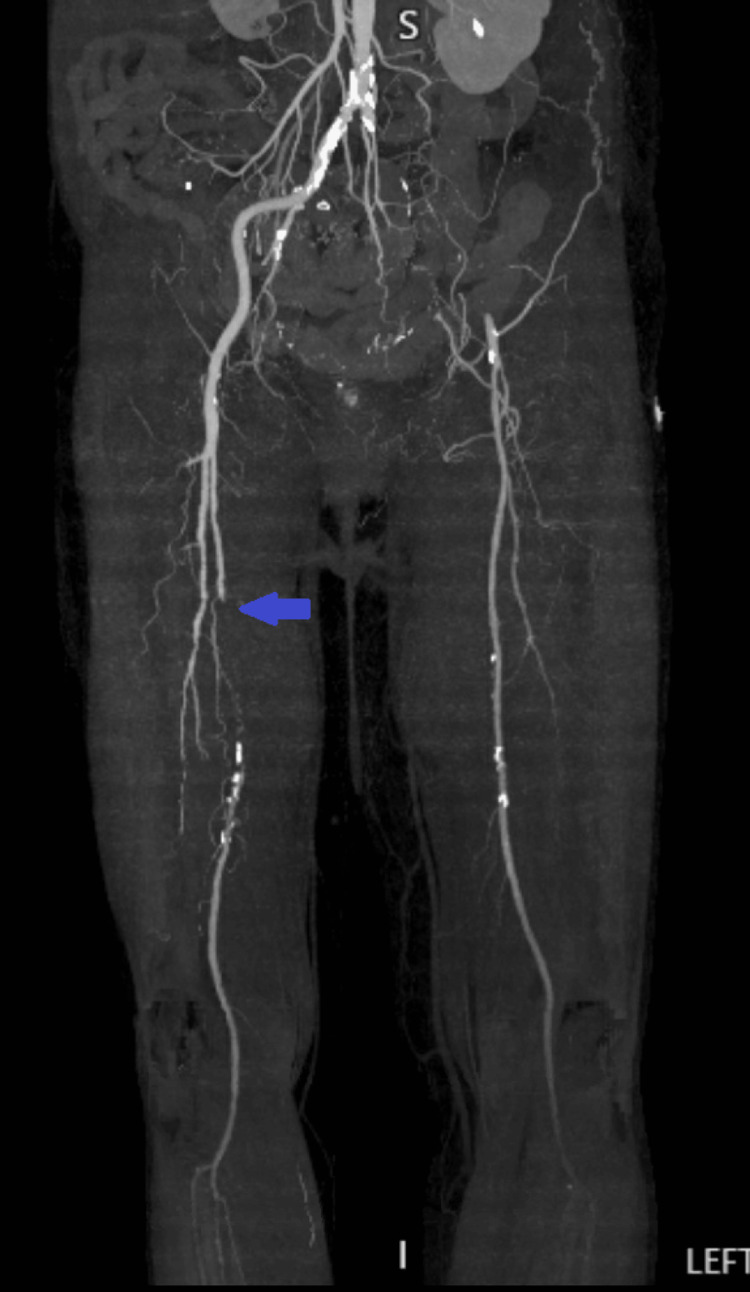
CT angiography imaging demonstrating occlusion of the right superficial femoral artery at the level of the arrow

After arriving at the tertiary care hospital, extensive imaging was performed. LLE venous imaging demonstrated totally occluding acute DVT present in the external iliac vein, common femoral vein, deep femoral vein, femoral vein, popliteal vein, posterior tibial vein, peroneal vein, and gastrocnemius vein. Computed tomography (CT) angiography showed focal ground glass densities in the lower lung fields, occlusion of the left common and external iliac arteries, occlusion of the left popliteal artery and distal arterial vasculature, and absent three-vessel runoff to the left foot with associated LLE subcutaneous soft tissue and muscular edema (Figure 2). CT imaging of the right lower extremity demonstrated occlusion of the right peroneal artery in the mid leg (Figure 3) and occlusion of the right superficial femoral artery (Figure 4). 

Additional laboratory testing performed demonstrated numerous derangements, including elevation of the following inflammatory markers - d-dimer, ferritin, c-reactive protein, lactate dehydrogenase, erythrocyte sedimentation rate, and creatine phosphokinase. The patient ultimately underwent successful mechanical thrombectomy of the left iliac vein thrombus with removal of an acute clot coupled with successful venoplasty and stenting without complication. She was medically cleared and discharged home on hospital day six with aspirin, Plavix, enoxaparin to warfarin bridge, and primary care and outpatient vascular surgery follow-up.

## Discussion

COVID-19 predisposes the infected individual to a hypercoagulable state resulting in both venous and arterial thrombosis with venous lesions being the most common. COVID-19 infection leads to a prolific inflammatory and systemic response culminating in microvascular changes, deposition of thrombotic factors in the micro-circulation, and endothelial injury. During this process, the inherent anticoagulation properties of endothelial cells are stifled, leading to cellular adhesion and vascular permeability which serves as a nidus for significant vascular occlusion [1,3]. It is for these physiologic reasons that the clinician must be acutely aware of COVID-19’s ability to manifest in a variety of ways, including lesser known sequela capable of catastrophic outcomes for the patient in question.

There are very few reports in the literature describing COVID-19 as the suspected etiology for concomitant arterial and venous thromboses, with the majority found in critically ill patients being treated in the intensive care unit (ICU) setting [3]. Of the cases reported, an overwhelming number are cited in patients with moderate to severe COVID-19 infection as there appears to be a correlation between severity of COVID-19 illness and multisystem organ injury and dysfunction. A report in the literature describes ALI in a patient being treated for COVID-19 with antibiotics and corticosteroids. This patient possessed significant systemic illness at the time of their ALI diagnosis and was notably hypotensive, tachycardiac, and hypoxic [4]. Another report in the literature describes ALI in a patient diagnosed with COVID-19 who was being treated in the ICU with enoxaparin, methylprednisolone, and intravenous tocilizumab for cytokine release syndrome in the setting of markedly elevated acute phase reactants, inflammatory markers, and deranged coagulation factors. On hospital day three, the patient was found to have severe arterial insufficiency after a blackened and hypothermic foot lacking peripheral pulses was discovered. The vascular surgery team determined that the limb was unsalvageable and an above-knee amputation was ultimately performed [5].

Our patient’s presentation is unique in that she presented with ALI in the setting of severe arterial occlusion and numerous totally occluding acute DVT despite being otherwise asymptomatic from a viral respiratory syndrome standpoint. Prompt recognition of our patient’s severely compromised extremity led to expedient transfer to a higher level of care which resulted in diagnostic imaging and surgical intervention ultimately yielding a positive outcome despite an initially poor prognosis with high mortality [5].

## Conclusions

ALI is a life-threatening, vascular emergency that must be rapidly recognized and treated as a means of preventing limb loss and death. Although COVID-19 is now well known to cause a hypercoagulable state leading to both venous and arterial compromise, it is critically important that the clinician is aware that the two entities can manifest concomitantly, particularly in an unsuspecting, asymptomatic patient with no risk factors for the diseases.

Our case underscores the need to expand the differential diagnosis and to consider ALI in patients infected with COVID-19 regardless of their symptomatology, particularly as atypical manifestations continue to be unearthed. Similarly, COVID-19 infection must be considered in the differential diagnosis and patients should be tested for the virus when presenting with clinical findings concerning for coagulopathic disease. A high index of suspicion for these disorders will serve to aid in early recognition, limb and life-saving treatment, and an overall decrease in morbidity and mortality.
